# Interplay between sympathetic nervous system and inflammation in aseptic loosening of hip joint replacement

**DOI:** 10.1038/s41598-018-33360-8

**Published:** 2018-10-30

**Authors:** M. Ribeiro-da-Silva, D. M. Vasconcelos, I. S. Alencastre, M. J. Oliveira, D. Linhares, N. Neves, G. Costa, R. Henrique, M. Lamghari, C. J. Alves

**Affiliations:** 1 Instituto de Investigação e Inovação em Saúde (i3S), Universidade do Porto, Porto, Portugal; 2 Instituto de Engenharia Biomédica (INEB), Universidade do Porto, Porto, Portugal; 3 Faculdade de Medicina, Universidade do Porto (FMUP), Porto, Portugal; 4 Serviço de Ortopedia e Traumatologia, Centro Hospitalar São João, Porto, Portugal; 5 Serviço de Anatomia Patológica & Grupo de Epigenética e Biologia do Cancro, Instituto Português de Oncologia do Porto, Porto, Portugal; 6 Instituto Ciências Biomédicas Abel Salazar (ICBAS), Universidade de Porto, Porto, Portugal

## Abstract

Inflammation is a common symptom in joint disorders such as rheumatoid arthritis, osteoarthritis (OA) and implant aseptic loosening (AL). The sympathetic nervous system is well known to play a critical role in regulating inflammatory conditions, and imbalanced sympathetic activity has been observed in rheumatoid arthritis. In AL it is not clear whether the sympathetic nervous system is altered. In this study we evaluated the systemic and local profile of neuroimmune molecules involved in the interplay between the sympathetic nervous system and the periprosthetic inflammation in hip AL. Our results showed that periprosthetic inflammation does not trigger a systemic response of the sympathetic nervous system, but is mirrored rather by the impairment of the sympathetic activity locally in the hip joint. Moreover, macrophages were identified as key players in the local regulation of inflammation by the sympathetic nervous system in a process that is implant debris-dependent and entails the reduction of both adrenergic and Neuropetide Y (NPY)-ergic activity. Additionally, our results showed a downregulation of semaphorin 3A (SEMA3A) that may be part of the mechanism sustaining the periprosthetic inflammation. Overall, the local sympathetic nervous system emerges as a putative target to mitigate the inflammatory response to debris release and extending the lifespan of orthopedic implants.

## Introduction

Osteoarthritis (OA) is one of the most prevalent chronic joint diseases and a major contributor to functional disability and loss of autonomy in older adults^1^. It is associated with a substantial economic and social burden, which will be even higher in the upcoming years, with the expected aging of the population^1^. Total joint replacement is considered the actual gold standard for the treatment of patients with severe OA, providing pain relieve, improving joint function and enhancing patients´ quality of life^2,3^. Unfortunately, total joint replacements can fail, mostly due to periprosthetic inflammation featured by sustained chronic inflammatory response initiated by implant degradation products that shed and accumulate in the neighbor tissue^4^. This adverse tissue reaction is orchestrated by a large plethora of immune cells of which the macrophage lineage has been shown to be of major relevance^4,5^. It is well established that macrophage differentiation towards M1 (pro-inflammatory) or M2 (anti-inflammatory) phenotype is of key importance to the inflammation state and/or resolution. *In vitro* studies have shown that Poly(methyl methacrylate) (PMMA) and ultra-high molecular weight polyethylene (UHMWPE) implant particles can polarize macrophages to pro-inflammatory M1 phenotype^6–8^. The activation of macrophages and other local cells results in the release of pro-inflammatory factors such as cytokines, chemokines, prostanoids, degradative enzymes and reactive oxygen species^5,9^. These factors underlie the chronic inflammatory scenario that may lead to painful synovitis, pathologic fracture of the surrounding bone and impaired function, instability and loosening of the implant^9^.

Over the past decades, accumulating evidence has clearly attributed a pivotal role to the sympathetic nervous system and its neurotransmitters in the regulation of chronic inflammatory conditions^10,11^. It has been demonstrated that the activation of the sympathetic nervous system in the context of inflammation results in the release of high amounts of sympathetic neurotransmitters known to induce an anti-inflammatory effect in a context-dependent manner^11,12^. The immunomodulatory effect of sympathetic nervous system can be achieved directly via adrenergic receptors (ADRs) expressed by the immune cells. Two types of ADRs have been characterized, the alpha (A) and beta (B), which were further divided into nine receptors subtypes (A1A, A1B, A1D, A2A, A2B, A2C, B1, B2 and B3)^13^. Stimulation of ADRB2 is reported to activate anti-inflammatory mechanisms on immune cells, while stimuli via ADRA activates pro-inflammatory mechanisms^14^. Therefore, the overall result will depend on the ADRs family being activated, which depends on the receptor expression profile and also on the norepinephrine concentration (norepinephrine has a high affinity to ADRA, only binding to ADRB when at high concentrations)^14^.

The neuropeptide Y (NPY), a neurotransmitter co-released with norepinephrine by the sympathetic nerve fibers, has also been reported to have modulatory effect on the activity of immune cells^15^. On the context of the immune response, of the five NPY receptors, the Y1 receptor (Y1R) is the most well studied, and has been shown to have a critical role in immunomodulation, as demonstrated by the attenuation of inflammation in Y1R knockout mice^16^. In healthy human joints, the synovium is highly innervated with both sympathetic and sensory nerve fibers^17^. In rheumatoid arthritis, data obtained in humans and in animal models revealed a deprivation of neuronal derived neurotransmitters in synovium tissue, due to the loss of sympathetic innervation^14,18–20^. Moreover, the extent of this deprivation is correlated with the severity of the inflammation. In fact, comparative studies reported a reduction of sympathetic innervation in synovial tissue of rheumatoid arthritis patients while in OA patients this does not occur^18^. In our previous work, we reported similar absence of sympathetic nerve fibers in periprosthetic tissues from AL patients while, again, in OA patients this does not occur^21^. In this line of evidence, the sympathetic activity is affected by the intensity of inflammation in the joint. However, it is still unknown whether in debris-associated periprosthetic inflammation occurs a complete shutdown of the sympathetic activity without any rescue mechanisms, and if the observed alterations are restricted to the joint or are also reflected at systemic level.

In this study we evaluated the systemic and the local profile of neuroimmune molecules involved in the interplay between sympathetic nervous system and the inflammatory response to the debris released by hip implants in AL. A comparison with OA was performed.

## Results

### Periprosthetic inflammation does not trigger the activation of the systemic neuroimmune regulatory pathway

**Figure 1:**
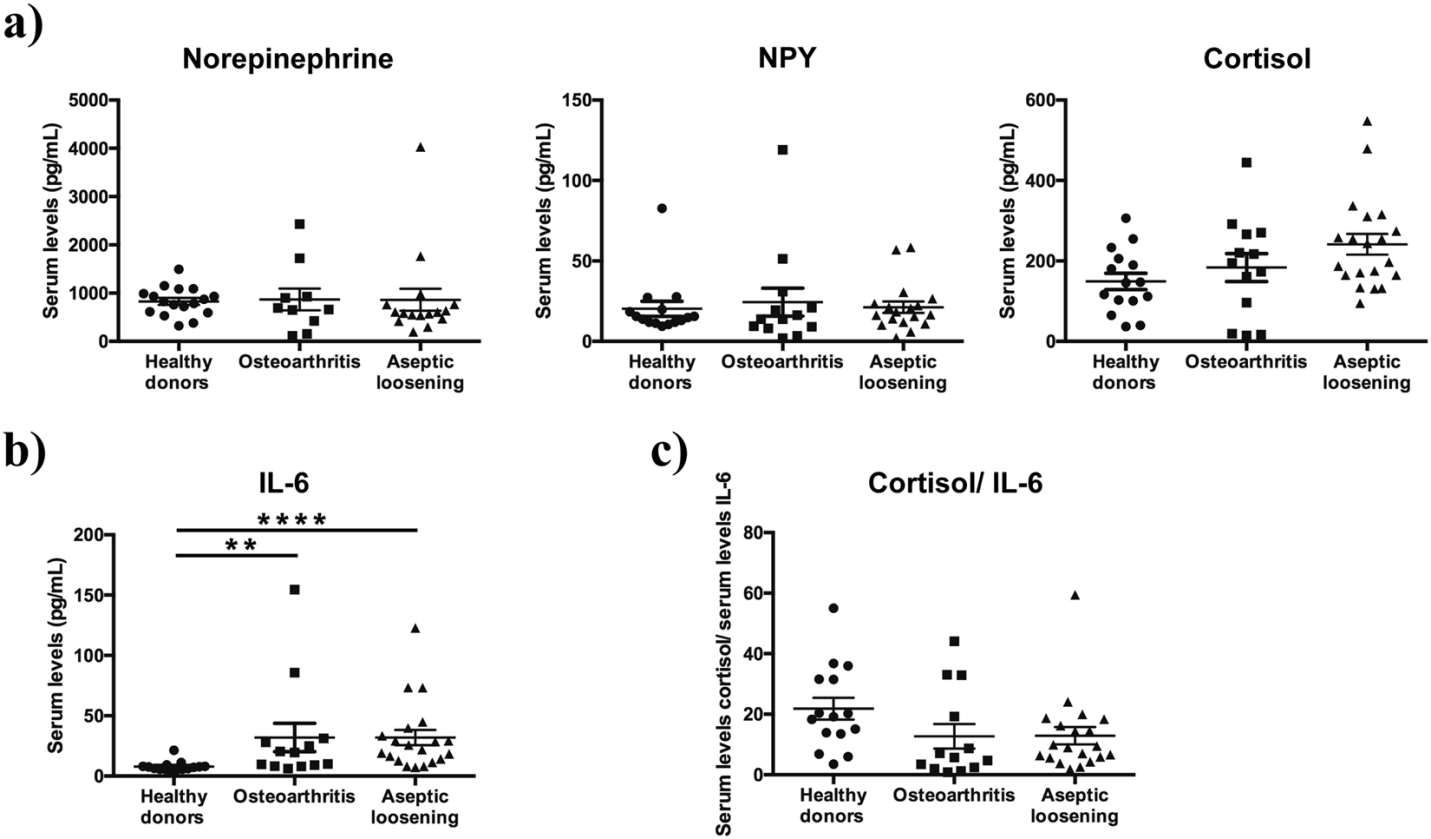
The systemic neuroimmune regulatory pathway was not targeted by periprosthetic inflammation in AL patients. The levels of norepinephrine, NPY and cortisol (**a**) and the levels of IL-6 (**b**) were evaluated by ELISA in the serum collected pre-operatively from OA and AL patients, and from healthy donors. The ratio serum cortisol/IL-6 was calculated (**c**). Results are presented as mean ± SEM, n = 13–15 for healthy donors and AO patients and n = 14–20 for AL patients. ^**^p < 0.01; ^****^p < 0.001.

Several studies have shown the activation of the systemic sympathetic nervous system in response to pro-inflammatory cytokines as a means to mobilize energy-rich molecules and sustain the inflammatory process^14^. In order to investigate the impact of periprosthetic inflammation on the systemic sympathetic nervous system activity, the serum levels of norepinephrine, epinephrine and NPY (markers of sympathetic nervous system activity) were measured in AL patients, OA patients, and healthy donors. In addition, the serum levels of cortisol were also assessed in the same groups as an indicator of the Hypothalamus-Pituitary-Adrenal (HPA) axis activity. The HPA axis together with the sympathetic nervous system compose the hormonal pathway through which the central nervous system exerts a regulatory control over inflammation^14^. No differences were found in the norepinephrine and NPY serum levels between the three groups (Fig. 1a). Epinephrine levels were below the detection limit of the used commercial kit. A tendency of higher cortisol levels was observed in AL patients when compared with healthy donors (p = 0.0658), although no statistically significant differences were found (Fig. 1a).

The serum levels of the pro-inflammatory cytokines IL-1β, IL-6 and TNF-α, key players in the activation of the sympathetic nervous system and HPA axis in response to inflammation^14^, were also measured in AL and OA patients and in healthy donors. The blood levels of IL-6 were higher in AL and OA patients than in healthy donors (*p* < 0.001 and *p* < 0.01, respectively) (Fig. 1b). The levels of IL-1β and TNF-α were observed to be below the ELISA kits’ detection limits in all groups.

Normal cortisol serum levels in the presence of high IL-6 concentrations are indicative of an inadequate cortisol secretion^22^, and the ratio serum cortisol/IL-6 was shown to be the more suitable indicator of the HPA axis activity^23^. Therefore, to further evaluate the HPA axis activity, the ratio of serum cortisol / IL-6 was calculated. Results showed no differences in this ratio between the groups, although a not statistically significant trend to lower values was observed in AO patients when compared with healthy donors (p = 0.0517) (Fig. 1c).

### Local sympathetic response is impaired in macrophages in periprosthetic tissues from AL patients

**Figure 2:**
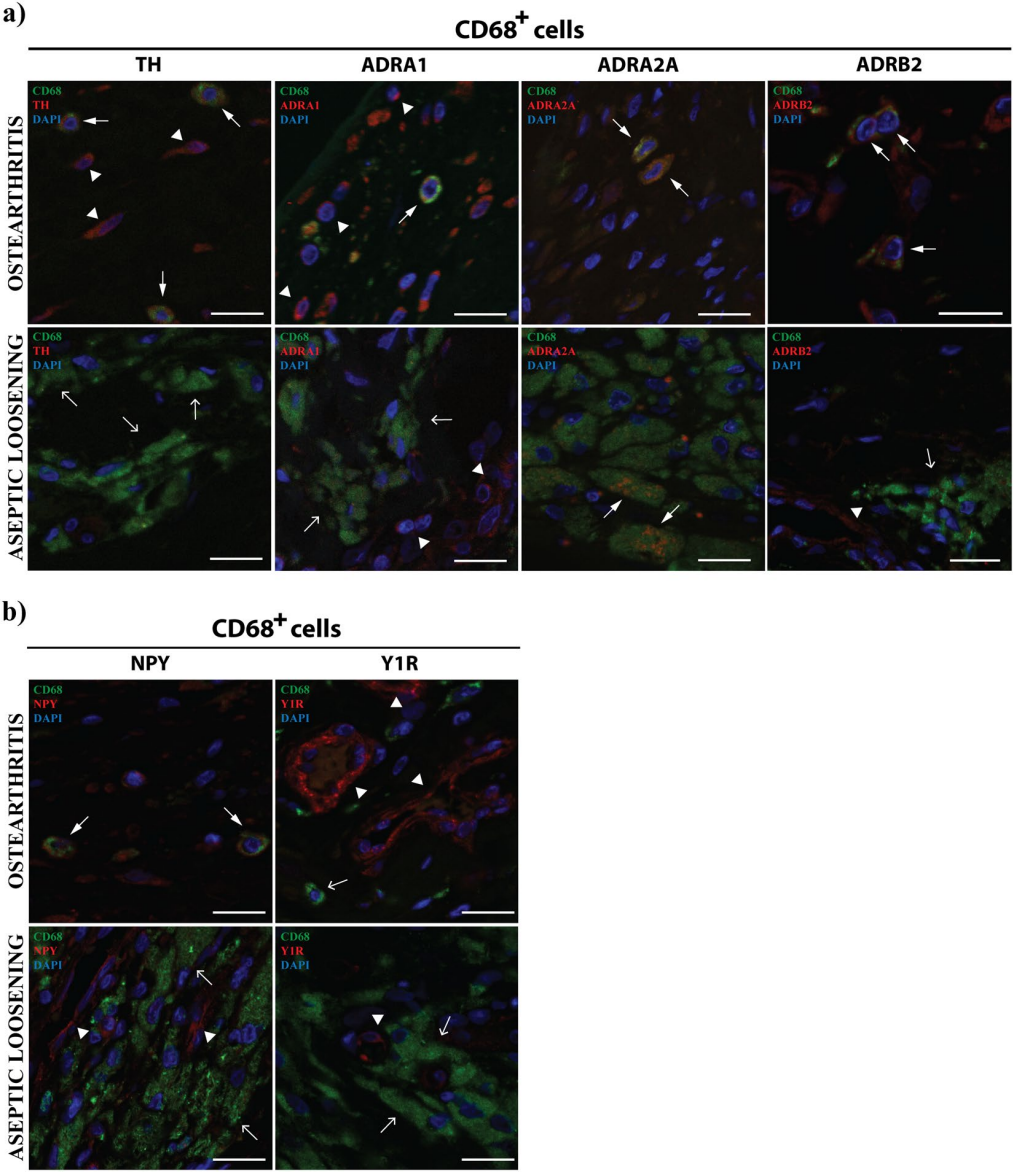
Macrophages in AL periprosthetic tissues do not express TH or ADRB2. The expression of TH, ADRA1, ADRA2A and ADRB2 (**a**) and the expression of NPY and Y1R (**b**) by macrophages (CD68^+^) was evaluated in periprosthetic tissues from AL patients and in synovial tissues from OA patients through double immunohistochemistry staining. Macrophages expressing TH, ADRA1, ADRA2A, ADRB2 or NPY are highlighted with triangle head white arrows. Simple head white arrows indicate macrophages and white arrowheads highlight TH, ADRA1, ADRB2, NPY and Y1R staining in cells other than macrophages (positive control). Scale bar = 20 μm.

Locally, the sympathetic nervous system is known to modulate the inflammatory response throughout adrenergic and NPY-ergic signaling^14^. In order to explore the effect of the sustained release of debris from implants on the local sympathetic immune-regulation, the expression of tyrosine hydroxylase (TH), ADRA1, ADRA2A, ADRB2, NPY and Y1R was analyzed in macrophages, B and T cells. A comparison between periprosthetic tissues and OA synovial tissues was performed. Macrophages in OA synovial tissues were found to express TH, ADRA1 and ADRB2, but not the macrophages present in periprosthetic tissues (Fig. 2a). ADRA2A was expressed by macrophages in both tissues (Fig. 2a). Macrophages in OA synovial tissues also stained positively for NPY but not macrophages in AL tissues (Fig. 2b).

**Figure 3:**
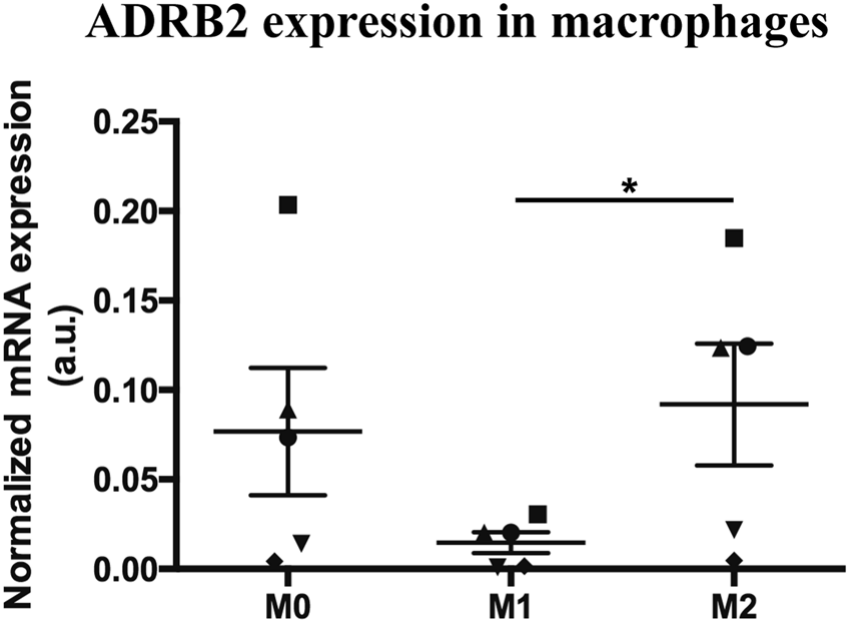
The *in vitro* expression of ADRB2 is lower in M1 as compared with M2 macrophages. The *in vitro* mRNA expression of ADRB2 was evaluated in M0, M1 and M2 macrophages phenotypes. Results are represented as mean ± SEM, for n = 5 per group. Each symbol represents macrophages obtained from one specific blood donor. ^*^p < 0.05.

Interestingly, the activation of ADRB2 in macrophages has been shown to promote a preferential differentiation of macrophages towards anti-inflammatory M2 phenotype over the pro-inflammatory M1 phenotype^24^. However, to our knowledge, the expression profile of ADRB2 in M1 and M2 macrophages was never described. Here we show that *in vitro* macrophages display a lower ADRB2 mRNA expression levels in M1 than in M2 phenotypes (p < 0.05) (Fig. 3). The *in vitro* analysis of ADRA1 and ADRA2 mRNA expression in M1 and M2 macrophages showed very low expression values, which were in some samples even below the detection limit. Still, from the results obtained, no differences were found in the ADRA1 and ADRA2 mRNA expression between M1 and M2 macrophages (data not shown).

**Figure 4:**
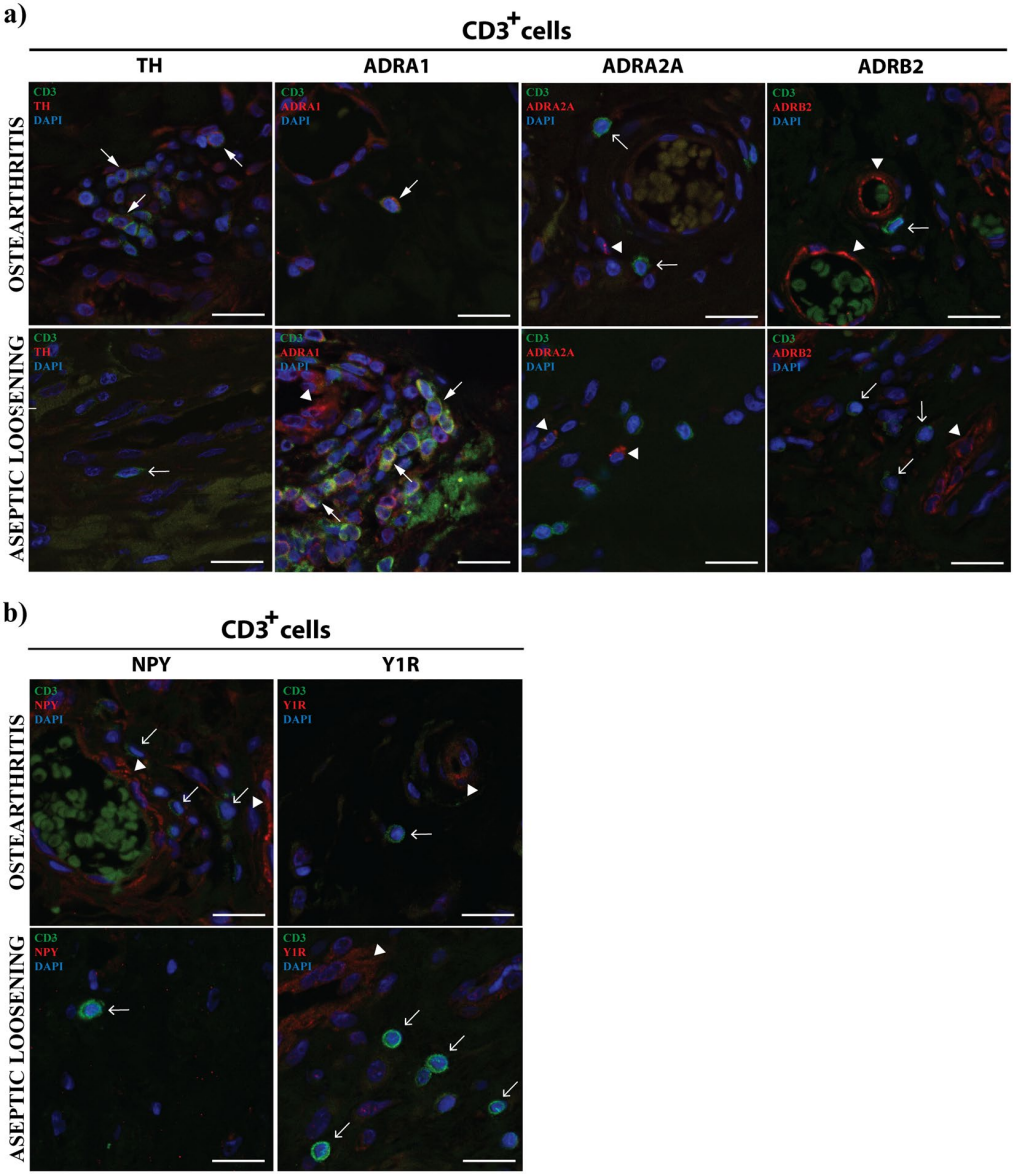
T cells express ADRA1 both in AL periprosthetic tissues and OA synovial membrane. The expression of TH, ADRA1, ADRA2A and ADRB2 (**a**), and the expression of NPY and Y1R (**b**) in T cells (CD3^+^) was evaluated in periprosthetic tissues from AL patients and in synovial tissues from OA patients through a double immunohistochemistry staining. T cells expressing TH or ADRA1 are highlighted with triangle head white arrows. Simple head white arrows indicate T cells and white arrowheads highlight ADRA1, ADRA2A, ADRB2, NPY and Y1R staining in cells other than T cells (positive control). Scale bar = 20 μm.

**Figure 5:**
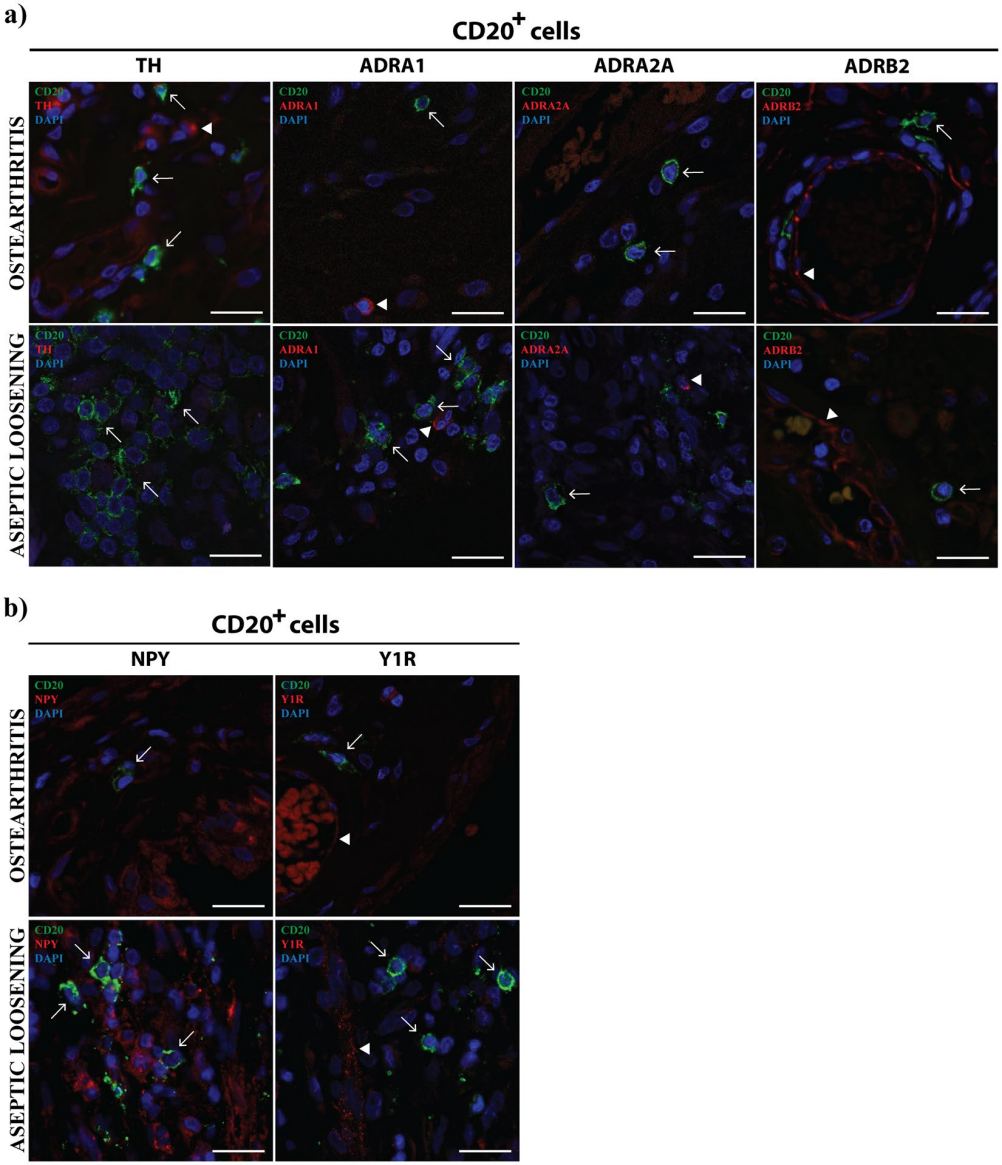
B cells in AL periprosthetic tissues and OA synovial membrane do not express TH, ADRA1, ADRA2A, ADRB2, NPY or Y1R. The expression of TH, ADRB2, ADRA1 and ADRA2A (**a**), and the expression of NPY and Y1R (**b**) in B cells (CD20^+^) was evaluated in periprosthetic tissues from AL patients and in synovial tissues from OA patients through double immunohistochemistry staining. White arrows indicate B cells and white arrowheads highlight positive staining for TH, ADRA1, ADRA2A, ADRB2 and Y1R in cells other than B cells (positive control). Scale bar = 20 μm.

T cells were found to express ADRA1 both in periprosthetic tissues and OA synovial membrane, but not ADRA2A and ADRB2 (Fig. 4a). T cells were also expressing TH in OA but not in AL tissues. B cells were not expressing the adrenergic markers investigated neither in AL nor in OA (Fig. 5a) and the expression of NPY and Y1R was absent from T and B cells in both pathologic conditions (Figs 4b and 5b).

### The absence of sympathetic innervation in periprosthetic tissues from AL patients is not mediated by the classic regulators of the innervation pattern

**Figure 6:**
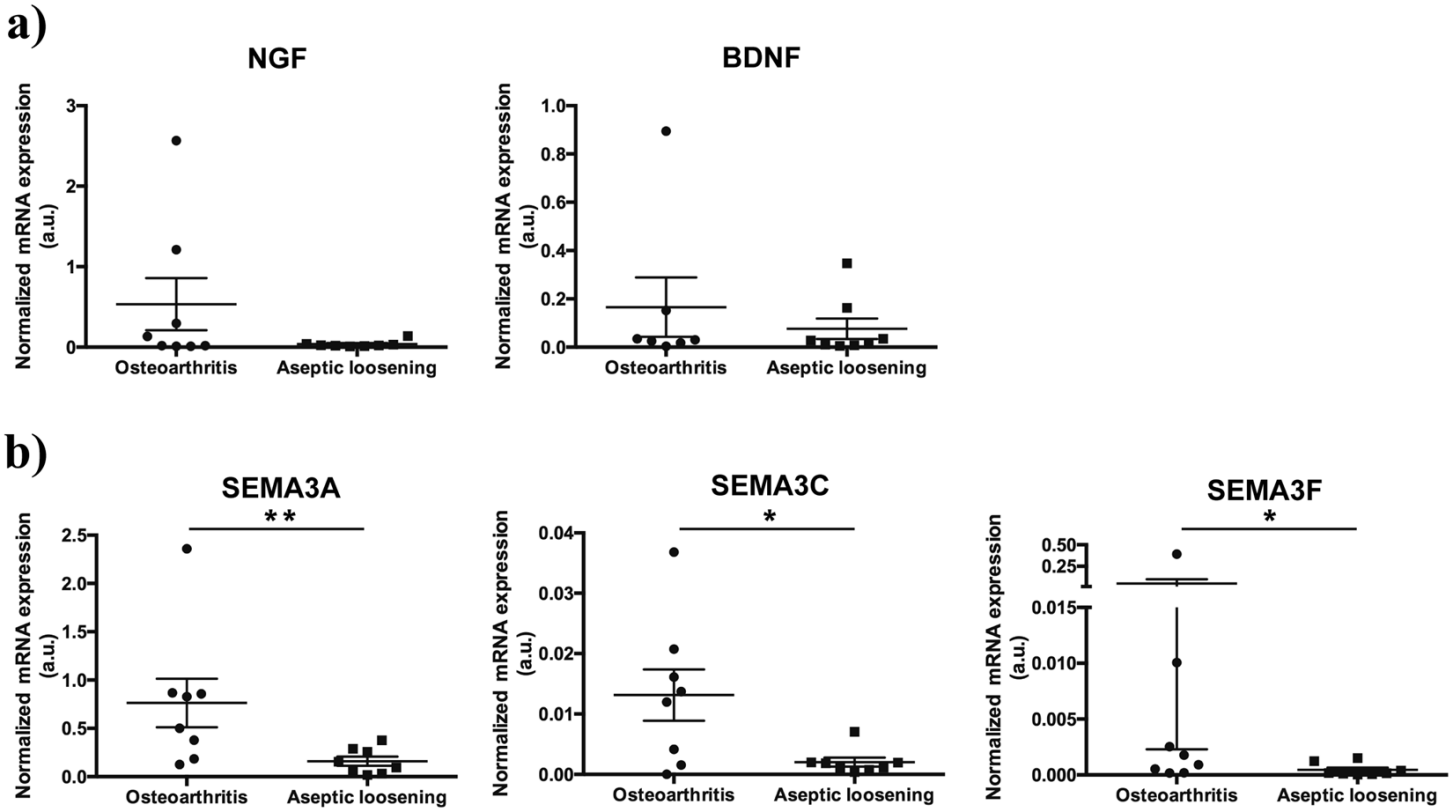
Neurotrophins and semaphorins expression in AL periprosthetic tissues and OA synovial membrane. The mRNA levels of neurotrophins NGF and BDNF (**a**), and semaphorins SEMA3A, SEMA3C and SEMA3F (**b**) were assessed in OA synovial membrane and periprosthetic tissues from AL patients. Results are represented as mean ± SEM, for n = 8–9 per group. ^**^p < 0.01, ^*^p < 0.05.

In our previous work^21^, using the same samples that were used in this study, we reported an unbalanced innervation pattern in hip periprosthetic tissues, mirrored by the lack of sympathetic nerve fibers. Here, in order to investigate the molecular mechanism underlying this effect, we evaluated the mRNA expression of the classical neurotrophins NGF and BDNF, of the nerve repellent molecule SEMA3A, and of the sympathetic nerve repellent factors SEMA3C and SEMA3F in the AL and OA joint tissues. No differences were found in the mRNA expression levels of NGF and BDNF between periprosthetic tissues and OA synovial membrane (Fig. 6a). Interestingly, the mRNA levels of SEMA3A, SEMA3C and SEMA3F were found to be decreased in periprosthetic tissues when compared with OA synovial tissues (SEMA3A: p < 0.01; SEMA3C and SEMA3F: p < 0.05) (Fig. 6b).

## Discussion

In this study we show that the periprosthetic inflammatory response is not decoded by the sympathetic nervous system at systemic levels, but is mirrored locally in the hip joint by the impairment of adrenergic and NPY-ergic activity in macrophages.

Pro-inflammatory cytokines such as TNF-α, IL-1β and IL-6 released by local immune cells were identified as key players in the central activation of sympathetic nervous system^14^. Moreover, implant debris were shown to promote the *in vitro* release of pro-inflammatory cytokines by lymphocytes^25^ and macrophages^26^, and in OA an increase of TNF-α in synovial membrane and blood, and of IL-6 in synovial fluid and blood was reported^27^. In this study, the comparison of the IL-1β, IL-6 and TNF-α profile showed an increase in the serum levels of IL-6 in both AL and OA patients (in comparison with healthy donors) but no differences between AL and OA. Such results highlight IL-6 as a main mediator of the systemic inflammatory response in the studied joint disorders. Still, these increased blood levels of IL-6 were not reflected in an increased systemic sympathetic tone neither in AL nor in OA patients, suggesting that the inflammatory response is not being regulated by the systemic sympathetic nervous system, even in scenarios where debris is being released. Moreover, the evaluation of the cortisol blood levels and the analysis of the cortisol/ serum IL-6 ratio, strong indicators of HPA axis activity^23^, showed no significant differences between the three analyzed groups, suggesting no impact in the HPA axis activity. The combined lack of activation of the systemic sympathetic nervous system and the HPA axis supports the absence of a systemic control of the inflammatory reaction in both OA and AL conditions.

Within the inflammation site, sympathetic nervous system can directly influence immune cells via the ADRs expressed on their cell surface^28^. The sympathetic immunomodulatory effects are known to be dependent on parameters such as the ADRs profile and the catecholamines concentration. The stimulation of ADRA, sensitive to low concentrations of norepinephrine, activates pro-inflammatory mechanisms, while ADRB2 activation by high concentrations of norepinephrine, triggers anti-inflammatory processes^14^. The sympathetic influence on immune function can also be exerted by other neurotransmitters co-released with norepinephrine, such as NPY^15^. In order to understand the impact of the debris release in the local sympathetic control of the inflammatory response we investigated both the adrenergic and the NPY-ergic activity in immune cells. In the follow up of our previous work^21^, the expression of TH, ADRA1, ADRA2A and ADRB2 (adrenergic markers) and of NPY and Y1R (NPY-ergic markers) in macrophages, B and T cells, the main immune cell types previously identified in AL periprosthetic tissues and in OA synovial membrane^21^, were investigated by immunohistochemistry.

Our immunostaining results showed that TH, ADRA1, ADRB2 and NPY were expressed by macrophages in OA synovial tissues but are absent in macrophages from periprosthetic tissues. These results show that both the adrenergic and the NPY-ergic arms are implicated in the local regulation of joint inflammation by the sympathetic nervous system in a debris-dependent manner and reinforce macrophages as key cellular players in the process. Moreover, the absence of TH and ADRB2 in AL macrophages suggests a lack of sympathetic-mediated anti-inflammatory mechanisms in response to periprostetic inflammation. It was demonstrated that the stimulation of ADRB2 in macrophages promotes their differentiation towards an M2 anti-inflammatory profile and serves as a mean to prevent hyper-inflammation^24^. In addition, the pro-inflammatory M1 macrophages are increased in AL periprosthetic tissues when compared with OA synovial membrane^6^, and *in vitro* studies showed that PMMA and UHMWPE implant particles promoted macrophages polarization towards a M1 pro-inflammatory phenotype^6–8^. In our study we observed that only OA macrophages, and not AL macrophages expressed ADRB2. Hence, we decided to assess whether macrophages polarization in M1 or M2 could impact the ADRB2 expression profile. For this aim, an *in vitro* experiment exploring ADRB2 expression in M1 and M2 macrophages was performed. The results showed that the expression of ADRB2 is lower in M1 pro-inflammatory phenotype than in M2 anti-inflammatory phenotype macrophages, suggesting that the lack of ADRB2 observed in macrophages in AL tissues might be involved in the harshness of the periprosthetic inflammation.

ADRA were also found to be differentially expressed in OA and AL macrophages (in OA both ADRA1 and ADRA2A are present while in AL only ADRA2A were found) suggesting a debris-dependent activity of ADRA-mediated macrophages pro-inflammatory mechanisms. Very limited research is available on ADRA expression profile and function in macrophages^29^. Whether such different expression profile is a direct consequence of the presence of debris, and/or conditions the different inflammatory outcomes is not known and should be further investigated.

Regarding NPY-ergic regulation, NPY produced by immune cells during inflammation is known to modulate the cellular activity through autocrine or paracrine actions^15,30^. NPY has been shown to decrease the expression of the pro-inflammatory TNF-α by macrophages after stimulation with LPS^31^ and to increase the expression of the anti-inflammatory TGF-β1^32^, contributing to ameliorate the inflammatory response. Moreover, it was recently suggested that NPY promotes the differentiation of macrophages towards a M2 anti-inflammatory phenotype^33^. Based on this information, and in the same way to what was found for ADRB2, the observed lack of NPY expression in AL macrophages may induce a preferential differentiation towards a pro-inflammatory phenotype. Such increase in the M1/M2 ratio may underlie the perpetuation of inflammation and increased osteolysis leading to implant failure. Although several immunomodulatory roles of NPY were suggested to occur through Y1R^15^, the expression of this receptor was not observed neither in macrophages in periprosthetic tissues nor in OA synovial membrane, hence, other NPY receptors (namely Y2R and Y5R) might be involved in the NPY regulation of macrophages activity^30,34^.

In regard to the B and T cells, we have previously shown that they are present in low number in both AL and OA tissues, and no differences in their population densities between the two joint conditions were found^21^. The activity of B and T cells has been reported to be modulated by the sympathetic nervous system, mostly through the ADRB2^35^. The sympathetic modulatory effects seem to be dependent on (i) the activation and differentiation state of the cells, on (ii) the molecular signalling pathway activated, and on (iii) the cytokine microenvironment^35^. In this study, we found that T cells and B cells were not expressing ADRB2 neither in AL nor in OA tissues, suggesting an absence of ADRB2 modulation of these cells populations. Moreover, B cells were also not expressing any of the investigated ADRA neither in OA nor in AL tissues indicating that these receptors are not involved in the regulation of these cells activity by the sympathetic nervous system. It was found that ADRA1 is expressed in T cells, both in OA and AL. This receptor has been described as an inhibitor of T cells proliferation^36^. Through our results, that show the expression of ADRA1 by mature T cells (CD3^+^) in both AL and OA tissues, a different putative role of ADRA1 in T cells-mediated inflammation, other than the inhibition of proliferation, which is independent of particles released by the implants, is suggested. As observed in macrophages, T cells were found to express TH in OA but not in AL tissues, supporting the higher ability of non-neuronal synthesis of catecholamines in OA synovial membrane when compared to AL periprosthetic tissues.

Evidence showed a repulsion of sympathetic nerve fibers from inflamed tissues that cause the loss of anti-inflammatory neurotransmitters and allow the establishment of a privileged pro-inflammatory area^18,37–39^. In a previous work, performed in the same samples used in the present study, we have also reported the repulsion of sympathetic innervation from periprosthetic tissues in AL patient but not from synovial membrane in OA patients^21^. Here, we investigate the putative involvement of well-studied neurotrophic factors, NGF and BDNF, of the nerve repellent molecule SEMA3A, and of the sympathetic nerve repellent factors SEMA3C and SEMA3F in this specific innervation pattern. No differences were found in the expression levels of NGF and BDNF between AL periprosthetic tissues and OA synovial membrane. The expression of the chemorepellent molecule SEMA3A, SEMA3C and SEMA3F was lower in periprosthetic tissues when compared with OA synovial membrane. Such results indicate that the most well studied molecules involved in the modulation of the innervation pattern were not responsible for the repulsion of the sympathetic innervation from periprosthetic tissues of AL patients. Considering that SEMA3A has also been described as having an immunomodulatory role^40–42^, the observed decrease SEMA3A expression may be part of the mechanism underlying the different inflammatory profile between OA synovial membrane and periprosthetic tissues. Actually, decreased levels of SEMA3A were observed in synovial tissues from rheumatoid arthritis patients compared to synovial tissues in OA patients, and the reduction of SEMA3A expression has been correlated with rheumatoid arthritis exacerbation^43^. Moreover, overexpression of SEMA3A reduced inflammation in a mouse model of collagen-induced arthritis^44^, further supporting SEMA3A as an important immunomodulatory molecule.

Overall, in this work we show that periprosthetic inflammation in AL does not trigger a systemic response of the sympathetic nervous system but impairs the local sympathetic activity as a putative mean to enable the perpetuation of the inflammatory state. Macrophages were highlighted as key cellular players in the local regulation of inflammation by the sympathetic nervous system, in a process that is implant debris-dependent and entails the reduction of the activity of both the adrenergic and the NPY-ergic systems. The absence of TH, ADRB2 and NPY expression in macrophages in periprosthetic tissues from AL patients may underlie a preferential differentiation of macrophages towards a M1 pro-inflammatory phenotype, promoting inflammation and increased osteolysis that may lead to implant failure. The local sympathetic nervous system emerges, therefore, as a putative target to mitigate the inflammatory response to debris release and extending the lifespan of orthopedic implants.

## Methods

### Samples

Biological samples were collected from three groups of patients. Group 1 was constituted by twenty patients (20 hips) undergoing hip revision surgery due to AL, after exclusion of infection, recurrent dislocation and periprosthetic fractures. All patients had a metal-on-polyethylene coupling and eleven prosthesis were cemented. All acetabular components were revised and in five of those the femoral stem was also exchanged. In four patients metallosis was observed and the mean time to revision was 120.05 ± 65.8 months. Group 2 was constituted by fifteen patients (15 hips), submitted to primary hip replacement surgery for primary OA, after excluding patients with a known history of inflammatory or neoplastic diseases. Group 3 was constituted by 15 healthy volunteers (i.e. without known osteoarticular or systemic diseases). Demographics from Groups 1–3 are presented in Table 1.

**Table 1:** Demographic data on included patients.

	N	Age (years)		Gender	
		Mean (±SD)	p	N of male/female	p
Group 1 Revision Surgery due to AL	20	70.35 ± 11.4	0.119	6/14	0.537
Group 2 Primary Surgery due to OA	15	63.33 ± 14.5		6/9	
Group 3 Healthy Donors	15	32.87 ± 3.4	< 0.001^*^	9/6	

AL- Aseptic loosening; OA- Osteoarthritis. ^*^Comparing Group 3 with both Group 1 and Group 2.

Serum was obtained from the blood collected, during the morning, previously to surgery in Groups 1 and 2, and in Group 3, and kept at −80 °C until analyses. Synovial tissue was removed during the surgical procedure in Group 2 patients, and periprosthetic tissue was collected in Group 1.

This study was approved by the Ethics Committee of Centro Hospitalar São João and all patients signed an informed consent to the use of their samples for research purposes. All the procedures were in accordance with the Helsinki Declaration of 1975, as revised in 2000.

### ELISA

The levels of Epinephrine/Norepinephrine, NPY and Cortisol were measured in the serum obtained from AL patients, OA patients and healthy donors, using ELISA kits from Abcam plc (Cambridge, UK; Catalog Number: AB108665), Merck KGaA (Darmstadt, Germany; Catalog Number: EZHNPY-25K) and Abnova (Taoyuan, Taiwan; Catalog Number: KA1877) respectively, according to the manufacturers’ protocols. The serum levels of interleukin (IL)-1β, IL-6 and Tumor necrosis factor alpha (TNF-α) were also measured in the same groups, using ELISA kits from BioLegend (CA, USA; Catalog Number: 430507).

### Monocyte isolation and macrophage differentiation

Human monocytes were isolated from healthy blood donors and differentiated into macrophages^45^. Briefly, 10^6^ monocytes/mL/3,8 cm^2^ were cultured for 10 days in RPMI1640 medium, supplemented with 10% Fetal bovine serum (FBS) and 100 U/mL penicillin and 100 μg/mL streptomycin, in the absence of Macrophages colony-stimulating factor (M-CSF) or other exogenous factors. 10 ng/mL LPS (Sigma-Aldrich) or IL-10 (ImmunoTools, Friesoythe, Germany) were added, for additional 72 h, to polarize macrophages towards M1 or M2 phenotype, respectively. Unstimulated macrophages (M0) were maintained with renewed medium.

### Double immunofluorescence staining

The expression of markers of the sympathetic nervous system by immune cells (macrophages, B cells and T cells) was evaluated through a double immunofluorescence staining analysis. The collected tissues were formaldehyde-fixed and processed for paraffin embedding, and cross-sections of 3 μm thickness were cut in the microtome (RM2255, Leica Biosystems). Deparaffinized and dehydrated sections were placed in antigen retrieval for 20 min at 97 °C (10 mM citrate buffer, pH 6.0). After quenching endogenous fluorescence with 0.1% NaBH_4_ and 100 mM NH_4_Cl, sections were incubated with blocking buffer (10% FBS, 1% Bovine serum albumin (BSA), 0.2% Triton X-100).

Simultaneous incubation of each primary antibody against immune cells (antibodies mouse anti-CD68 (clone 514H12, dilution 1:100, Novocastra, UK), anti-CD20 (clone L26, dilution 1:100, Cell Marque, USA), anti-CD3 (clone PS1, dilution 1:100, Biocare Medical, USA)) with each primary antibody against sympathetic markers (antibodies rabbit anti-TH (dilution 1:100, Merck KGaA, Darmstadt, Germany), anti-ADRA1 (dilution 1:100, Abcam, USA), anti-ADRA2A (dilution 1:200, Abcam, USA), anti-ADRB2 (dilution 1:100, Proteintech, USA), anti-NPY (dilution 1:1000, Sigma-Aldrich, USA) or anti-Y1R (dilution 1:500, Immunostar, USA)) were performed overnight at 4 °C.

For signal detection, tissue sections were incubated for 1 hour at room temperature (RT) with a mixture of anti-rabbit Alexa Fluor 568 antibody and anti-mouse Alexa Fluor 488 antibody (1:1000 dilution, Life Technologies, USA), incubated with DAPI and then mounted with Fluoroshield Mounting Medium (Abcam, USA). Immunostaining images were acquired on the confocal Leica TCS SP2 AOBS (Leica Microsystems, Germany) and Leica TCS SP5 microscope (Leica Microsystems, Germany).

### Gene expression analysis

Synovial tissues were homogenized in liquid nitrogen using a mortar and pestle to preserve RNA integrity. RNA from synovial tissues and from macrophages was extracted and purified using TRIzol (Invitrogen, UK) and Direct-zol™ RNA MiniPrep (ZYMO Research, USA), according to the manufacturers’ instructions.

RNA purity was estimated from the ratio of absorbance readings at 260 and 280 nm and only ratio between 1.8 and 2 were accepted. RNA quality was verified in agarose gel and RNA concentration was determined in a NanoDrop spectrophotometer (NanoDrop™ 1000 Spectrophotometer, Thermo Fisher Scientific, Wilmington, Delaware, USA NanoDrop). RNA was reverse transcribed using the SuperScript^TM^ First-Strand Synthesis System for reverse transcription-polymerase chain reaction (RT-PCR) (Invitrogen, Carlsbad, CA, USA).

The transcriptional levels of ADRA1A, ADRA1B, ADRA1D, ADRA2A and ADRB2 in macrophages and neurotrophins (Nerve growth factor (NGF) and Brain-derived neurotrophic factor (BDNF)) and semaphorins (SEMA3A, SEMA3C and SEMA3F) in the AL and OA tissues were evaluated by quantitative real time PCR (qRT-PCR) in the CFX96 Touch Detection System (Bio-rad, USA). β2 microglobulin (B2M) was used as reference gene for internal normalization. The primers used were as follows: ADRA1A sense primer: 5′-TCATGTACTGCCGCGTCTAC-3′; ADRA1A antisense primer: 5′-GGGCGTTTTTCCGATGGATG-3′; ADRA1B sense primer: 5′-CTCTACCGCTTGGCTCCTTGT-3′; ADRA1B antisense primer: 5′-GGAGCATGGGTAGATGATGGG-3′; ADRA1D sense primer: 5′-TCTCCCGTGAGAAGAAAGCG-3′; ADRA1D antisense primer: 5′-CGGGAACAAGGAGCCGAG-3′; ADRA2A sense primer: 5′-ATCCTGGCCTTGGGAGAGAT-3′; ADRA2A antisense primer: 5′-TCTCAAAGCAGGTCCGTGTC-3′; ADRB2 sense primer: 5′GGACTTCCATTGATGTGCTGT-3′; ADRB2 antisense primer: 5′-GTCAGCAGGCTCTGGTACTTG-3′; NGF sense primer: 5′-AGCGCAGCGAGTTTTGG-3′; NGF antisense primer: 5′-GCTGCTCCCTTGGTAAACTG-3′; BDNF sense primer: 5′- GATGCTCAGTAGTCAAGTGCC-3′; BDNF antisense primer: 5′-GCCGTTACCCACTCACTAATAC-3′; SEMA3A sense primer: 5′CAGCCATGTACAACCCAGTG-3′; SEMA3A antisense primer: 5′-ACGGTTCCAACATCTGTTCC-3′; SEMA3C sense primer: 5′-ATCCGGTCCTGATCTTCATC-3′; SEMA3C antisense primer: 5′-CAGCCCCAAGCAAGAGTTTA-3′; SEMA3F sense primer: 5′-CCAACTACCAGTGGATGCCC-3′; SEMA3F antisense primer: 5′-GTACACGGCCTGGTACATGA-3′; B2M sense primer: 5′-CCAGCGTACTCCAAAGATTCAG-3′; B2M antisense primer: 5′- AGTCAACTTCAATGTCGGATGG-3′. Relative transcription levels were calculated by comparative threshold cycle quantification (ΔC_t_ method) using B2M as reference gene.

### Statistical analyses

All data were assessed for normal distribution and non-parametric analyses were performed whenever normal distribution was not followed. Hormones and IL-6 levels, and the cortisol/IL-6 ratio were analyzed by Kruskal-Wallis test followed by Dunn’s multiple comparison test. mRNA expression of neutrophins and semaphorins in the synovial tissues was analyzed by Mann-Whitney test and ADRs mRNA expression in macrophages by the Repeated Measures ANOVA followed by Holm-Sidak’s multiple comparisons test. Differences were considered at the significant level of p < 0.05. All data are expressed as mean ± SEM. Statistical analyses were performed using the software Prism 6 (GraphPad software, San Diego, CA, USA).
